# Prenatal Diagnosis of Fetal Peters' Plus Syndrome: A Case Report

**DOI:** 10.1155/2013/364529

**Published:** 2013-07-29

**Authors:** Neerja Gupta, Anita Kaul, Madhulika Kabra

**Affiliations:** ^1^Division of Genetics, Department of Pediatrics, All India Institute of Medical Sciences, New Delhi 110029, India; ^2^Fetal Medicine Unit, Indraprastha Apollo Hospital, New Delhi, India

## Abstract

Peters' plus syndrome is a rare but clinically recognizable autosomal recessive ocular genetic syndrome. Diagnosis during the fetal life is challenging due to the presence of nonspecific findings such as ventriculomegaly in the growth-retarded fetuses. We report the first case of fetal Peters' plus syndrome from India, where fetal ultrasound and the family history were helpful in providing a clue to the diagnosis that was confirmed later on by the DNA analysis.

## 1. Introduction

Peters'-plus syndrome also known as the Kivlin-Krause syndrome (OMIM number 261540) is a rare autosomal recessive congenital disorder of glycosylation [1]. Exact incidence is not known. Fewer than 75 cases with this condition have been reported worldwide. Peters' plus syndrome can be clinically recognized by the presence of typical Peters' anomaly (an anterior segment abnormality due to developmental dysgenesis causing central corneal opacity) along with facial dysmorphism, cleft lip or palate, disproportionate short stature, and varying degree of intellectual disability. Dysmorphic features include a round face, hypertelorism, short palpebral fissures, prominent forehead, cupid bow lip, thin upper lip, long philtrum, with or without cleft lip and/or cleft palate, hearing loss, and joint hyperextensibility. Presence of congenital heart defects, genitourinary anomalies such as hydronephrosis, hypospadias, cryptorchidism, hypoplastic clitoris, and labia majora, and structural brain malformations may alter the prognosis [2]. Lesnik Oberstein et al. [3] showed that homozygosity for loss-of-function mutations in beta 1, 3-galactosyltransferase-like gene (*B3GALTL*) on chromosome 13 (13q12.3) is responsible for this phenotype. The majority of the patients have homozygous hot spot splice mutation in intron 8 (c.660+1G>A), causing a defective glycosylation of thrombospondin type 1 repeats [1]. Peters' anomaly with multiple congenital anomalies has also been reported in few patients with unbalanced chromosomal abnormalities such as 11q interstitial deletion, ring chromosome 21, partial trisomy 5p, partial monosomy 4q, and varying-size deletions (781 kb–~1.5 Mb) on microarray involving one of the parental alleles and a pathogenic point mutation on the other allele [3, 4]. Sonographic diagnosis during the fetal life is challenging due to the presence of nonspecific findings such as ventriculomegaly in the growth-retarded fetuses which could be a part of multiple genetic syndromes. We report the first case of fetal Peters'-plus syndrome from India. The diagnosis was suspected on the detailed fetal evaluation and a positive family history and confirmed later on by molecular testing.

## 2. Case Presentation

**Figure 1:**
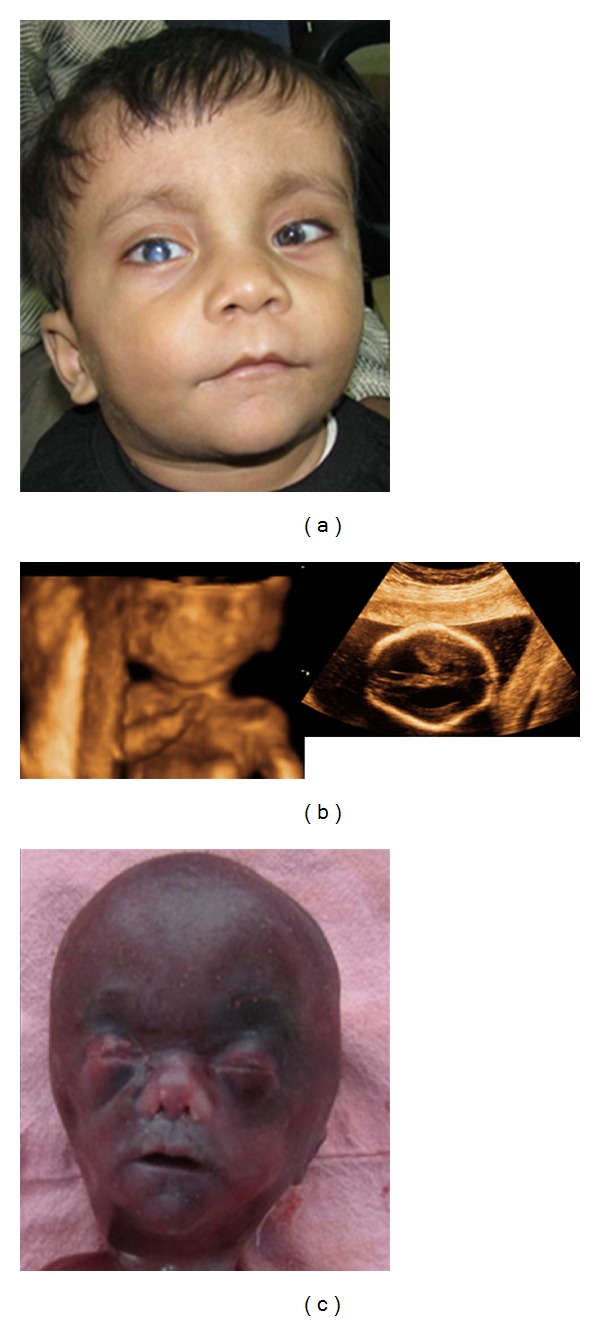
(a) Proband with Peters' anomaly and facial dysmorphism. (b) Ultrasound showing, long philtrum, short nose and ventriculomegaly. (c) Fetus with facial dysmorphism. Note prominent forehead, hypertelorism, long philtrum, short nose, anteverted nostrils, and thin upper lip.

A 23-year-old third gravida lady was referred at 19-week gestation for genetic counseling for having a previous 3-year-old male child with short stature and developmental delay. She also had a history of male intrauterine death at 32 weeks. The proband was a product of third-degree consanguineous marriage and was born at full term at home. Exact anthropometric details at birth were not available. He was diagnosed to have bilateral Peters' anomaly with secondary glaucoma at the age of 2 months. He underwent corneal transplant and right-sided iridotomy at the age of 1 year. He had a mild developmental delay. Examination showed the presence of proportionate short stature, height measuring 73 cm (<3rd centile), weight 10 kg (<3rd centile), and head circumference 47 cm (at 3rd centile). He had a round face, long philtrum, short nose, and large cupid bow mouth along with brachydactyly and small terminal phalanges (Figure 1(a)). Computerized tomography for brain and echocardiography were normal. A provisional diagnosis of the Peters' plus syndrome was made and DNA was stored for subsequent molecular testing for *B3GALTL* gene. During the current pregnancy, a targeted high-resolution fetal ultrasound showed the presence of bilateral mild ventriculomegaly (Figure 1(b)), short long bones (all long bones below 5th centile), brachycephaly, single umbilical artery, long philtrum (Figure 1(b)), low set ears, and clinodactyly. Ultrasound examination of eyes was normal. In view of these findings and the family history, a probable diagnosis of Peters' plus syndrome in the fetus was made and the couple was counseled. The couple opted for the termination of pregnancy. Fetal samples were preserved for DNA as well as the chromosomal analysis. Fetal examination confirmed the ultrasound findings but also revealed some additional findings such as the presence of prominent forehead, hypertelorism, thin upper lip, anteverted nostrils, brachydactyly (Figure 1(c)), and a caudal appendage. 

**Figure 2:**
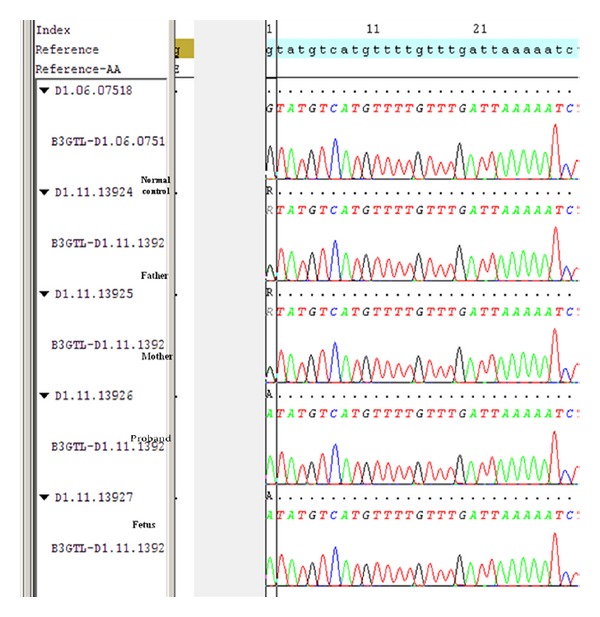
It shows electropherogram of father, mother, proband, and the fetus. Reference is the reference sequence of the *B3GALTL* gene. The region shown is the beginning of intron 8.

Chromosome analysis from fetus and proband was normal. Fetal DNA and the DNA from the proband and parents blood were obtained using standard protocols. *B3GALTL* exons and flanking introns were amplified by PCR. Direct sequencing of the PCR products was performed using ABI 3730 Sequencer. Both proband and the fetus had homozygous c.660+1G>A splice site mutation in intron 8 and the parents were heterozygous carriers for the same mutation (Figure 2).

## 3. Discussion

Peters' plus syndrome is an ocular genetic disorder due to a defect in *B3GALTL* gene causing Peters' anomaly along with other multisystem abnormalities. Although it is easier to make a postnatal diagnosis, prenatal diagnosis of fetal Peters' plus syndrome based solely on ultrasound findings may be difficult due to the presence of variable and nonspecific findings. Few ultrasound features like hydrocephalus, agenesis of corpus callosum, microphthalmia and cleft lip/palate, and other structural anomalies in a growth-retarded fetus can point towards a probable diagnosis of fetal Peters' plus syndrome [5, 6], especially in the presence of a positive family history. In the present case, fetus was suspected to have Peters' plus syndrome due to the presence of an affected sib and the supporting ultrasound dysmorphic features such as bilateral mild ventriculomegaly, short long bones (all long bones below 5th centile), a long philtrum, and clinodactyly as seen in the proband. There was no eye abnormality in the fetus at 19-week gestation and this could be explained by the intrafamilial heterogeneity [5]. A confirmatory diagnosis was possible only after posttermination fetal examination and through DNA analysis that showed the most frequent previously described mutation, both in the fetus and the proband, explaining the typical phenotype associated with this mutation.

We report this case as it reiterates the usefulness of careful family history and thorough dysmorphologic evaluation of the index case as well as the fetus. High-resolution ultrasound is helpful in identifying some of the nonspecific structural abnormalities such as ventriculomegaly and facial dysmorphism associated with such clinically heterogeneous disorder. Nevertheless, a confirmed diagnosis by molecular methods is the most reassuring both for the family and the physicians. It also provides them with the option for early prenatal diagnosis in subsequent pregnancies, much prior to the appearance of nonspecific ultrasound abnormalities.
